# Fluorescent carbon dots as an efficient siRNA nanocarrier for its interference therapy in gastric cancer cells

**DOI:** 10.1186/s12951-014-0058-0

**Published:** 2014-12-30

**Authors:** Qing Wang, Chunlei Zhang, Guangxia Shen, Huiyang Liu, Hualin Fu, Daxiang Cui

**Affiliations:** School of Life Sciences and Biotechnology, Shanghai Jiao Tong University, Shanghai, 200240 China; Institute of Nano Biomedicine and Engineering, Key Laboratory for Thin Film and Microfabrication Technology of the Ministry of Education, Department of Instrument Science & Engineering, School of Electronic Information and Electrical Engineering, Shanghai Jiao Tong University, 800 Dongchuan RD, Shanghai, 200240 China

**Keywords:** Carbon dots, siRNA interference therapy, Gastric cancer, Nanocarriers

## Abstract

**Background:**

Fluorescent carbon dots (Cdots) have attracted increasing attention due to their potential applications in sensing, catalysis, and biomedicine. Currently, intensive research has been concentrated on the synthesis and imaging-guided therapy of these benign photoluminescent materials. Meanwhile, Cdots have been explored as nonviral vector for nucleic acid or drug delivery by chemical modification on purpose.

**Results:**

We have developed a microwave assisted one-step synthesis of Cdots with citric acid as carbon source and tryptophan (Trp) as both nitrogen source and passivation agent. The Cdots with uniform size show superior water solubility, excellent biocompatibility, and high quantum yield. Afterwards, the PEI (polyethylenimine)-adsorbed Cdots nanoparticles (Cdots@PEI) were applied to deliver Survivin siRNA into human gastric cancer cell line MGC-803. The results have confirmed the nanocarrier exhibited excellent biocompatibility and a significant increase in cellular delivery of siRNA, inducing efficient knockdown for Survivin protein to 6.1%. In addition, PEI@Cdots complexes mediated Survivin silencing, the arrested cell cycle progression in G_1_ phase as well as cell apoptosis was observed.

**Conclusion:**

The Cdots-based and PEI-adsorbed complexes both as imaging agents and siRNA nanocarriers have been developed for Survivin siRNA delivery. And the results indicate that Cdots-based nanocarriers could be utilized in a broad range of siRNA delivery systems for cancer therapy.

**Electronic supplementary material:**

The online version of this article (doi:10.1186/s12951-014-0058-0) contains supplementary material, which is available to authorized users.

## Introduction

Over recent decades, great advances have been made in the combination of nanotechnology and medicine, which is paving the way towards the goal of clinic application [1,2]. A large amount of biocompatible fluorescence nanomaterials, such as quantum dots, metal nanoclusters, and fluorescent polymers, have been developed [3-5]. Carbon dots (Cdots) are currently emerging as a class of promising fluorescent probe on account of their low photobleaching, no optical blinking, tunable photoluminescence, versatile surfaces, and excellent biocompatibility [6,7]. Therefore, fluorescent Cdots possess additional benefits over organic fluorophores and semiconductor quantum dot, which are more or less circumscribed by their photobleaching or intrinsic potential hazards of heavy metal elements (e.g. Cd and Pb) [8]. These excellent properties of Cdots have made bright prospects in the applications of bioimaging, drug delivery, biochemical detection, and sensors [9-11]. Currently, intensive research has been focus on the synthesis of Cdots with high quantum efficiency and the construction of multifunctional systems based on Cdots [10,12,13]. Until now, various precursors including graphite, C_60_, citric acid, glucose, and silk have been developed for the preparation of Cdots with a wide variety of approaches, processes, and tools [10,12,14-16]. Sun’s group has reported a new strategy to prepare core-shell dots based on Cdots doped with inorganic salts with quantum yield around 45% ~ 60%, but the preparation process is quite complicated [17].

Recently, our group has developed a green synthetic route for Cdots with high quantum yield around 24.2% using Ribonuclease A (RNase A) as an assisting and passivating reagent *via* microwave assisted one step procedure [18]. Interestingly, the RNase A@Cdots can effectively inhibit the survival rate of cancer cells. But the price of RNase A is not economic enough. Taking into account the mechanism of the photoluminescence (PL) enhancement in RNase A@Cdots, electron-donating effect from neighbor amino acids especially those with benzene rings could play an important role. Therefore, we select tryptophan (Trp), a kind of amino acids with benzene ring while possesses a higher nitrogen content than tyrosine and phenylalanine, for the synthesis of Cdots with lower cost. Besides, we have ever developed a new theranostic platform based on photosensitizer-conjugated Cdots with excellent imaging and tumor homing ability for NIR fluorescence imaging guided photodynamic therapy [19]. However, till now few reports are closely associated with the using of Cdots as gene transfection vector for cancer therapy.

RNA interference (RNAi) has emerged as a valuable research tool to downregulate the expression of specific target proteins in a wide variety of cells [20]. RNAi is a biological process in which RNA molecules inhibit gene expression by causing the destruction of specific mRNA molecules. Viral delivery such as Lentivirus, Adenovirus and Adeno-Associated-Virus, has successfully been used for therapeutic applications, including cancer therapy [21,22]. To deal with concerns over the potential risk of undesired immune and toxic side reactions in virus-mediated nucleic acid delivery systems, non-viral gene delivery systems based on inorganic nanoparticles, cationic liposomes, and cationic polyamidoamine dendrimers have been employed as carriers for gene silencing [23-25]. The nanomaterials-based non-viral gene delivery systems have benefited from many advantages over viral vectors, as they are simple to prepare, rather stable, easy to modify and relatively safe. Meanwhile, Cdots would be an ideal nanocarrier due to the high biological safety, well-defined structures together with their tunable surface functionalities.

**Figure 1:**
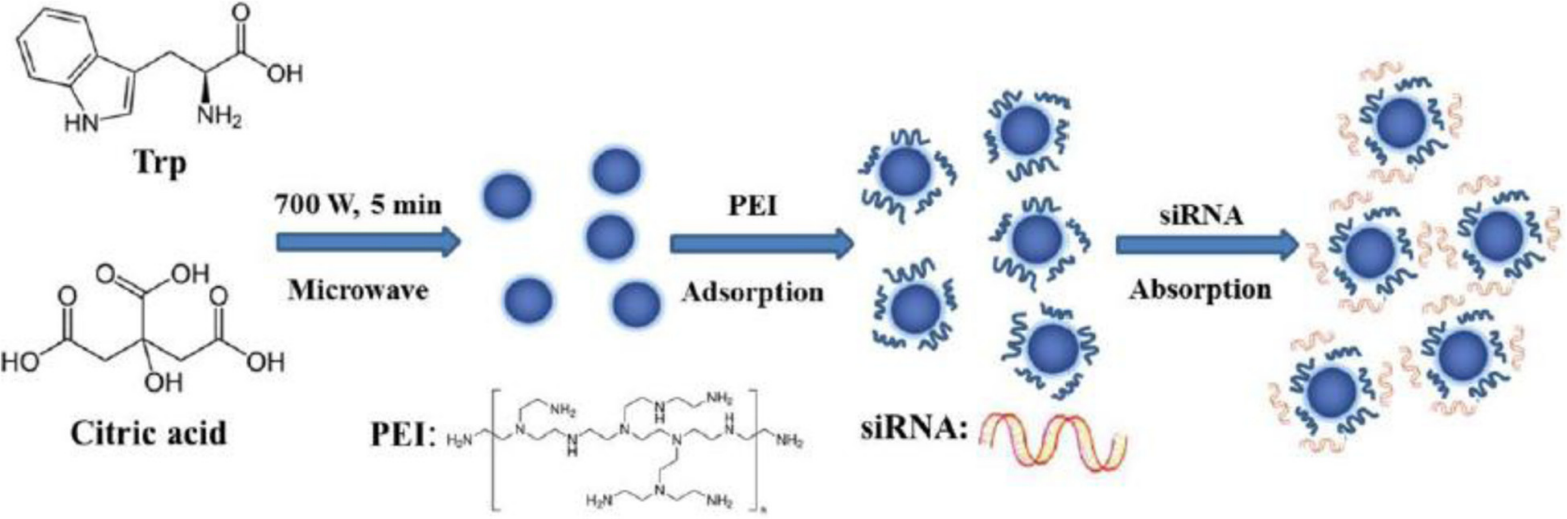
**Schematic illustration of the formation of Cdots and the Cdots-based nanocarrier for the delivery of siRNA.**

In this work, the Cdots-based and PEI-adsorbed nanocarrier (Cdots@PEI) was developed to delivery siRNA against antiapoptotic protein Survivin in human gastric cancer cell line MGC-803. It is well known that Survivin play an important role in cell division, apoptosis, and checkpoint mechanisms of genomic integrity [26,27]. In addition, Survivin expression is often upregulated in human cancers, as it can be treated as a specific tumor marker with prognostic and therapeutic implications from studies of gastric carcinomas [28]. For the preparation of the Cdots-based nanocarrier, firstly, the Cdots was directly synthesized *via* microwave pyrolysis of citric acid in the presence of tryptophan and purified by gradient centrifugation and dialyzed with MWCO 3500 dialysis bag for 72 h. Secondly, the Cdots@PEI complexes were formed by electrostatic interaction between negatively charged Cdots and positively charged PEI (as depicted in Figure 1). The Cdots@PEI complexes were used to mix with Survivin siRNA (siRNA-Cdots@PEI). Afterwards, we investigated gene transfection efficacy, cellular uptake, and biological effects of the siRNA-Cdots@PEI complexes towards gastric cancer cells MCG-803 by means of reverse transcription polymerase chain reaction (RT-PCR), Western blotting, apoptosis assay and cell cycle analysis. The results showed that siRNA can attach to the surface of the Cdots@PEI complexes and notably enhanced the gene delivery efficiency. The system renders the possibility of Cdots to serve as a universal transmembrane carrier for intracellular gene and drug delivery and imaging applications in cancer gene therapy.

## Experimental sections

### Materials

Citric acid, L-tryptophan (99%) and polyethylenimine (PEI) with a molecular weight of 1800 Da were purchased from Aladdin Reagent Co. Ltd. (Shanghai, China). Dimethyl sulfoxide (DMSO) was obtained from Sinopharm Chemical Reagent Co., Ltd, China. Hieff™ qPCR SYBR® Green Master Mix and Annexin V-FITC/PI Apoptosis Detection Kit were purchased from Yeasen Corporation (Shanghai, China). 3-[4, 5-dimethylthiazol-2yl]-2,5-diphenyltetrazolium bromide (MTT) was purchased from Invitrogen Corporation (Carlsbad, CA, USA). Random primer and M-MLV reverse transcriptase were purchased from Promega (Madison, WI, USA). RIPA lysis buffer and BCA (bicinchoninic acid) protein assay kit were purchased from Beyotime Biotechnology (Jiangsu, China). Monoclonal rabbit anti-Survivin antibody, polyclonal rabbit anti-β-actin antibody, and horseradish peroxidase (HRP)-conjugated goat anti-rabbit IgG were purchased from Epitomics (Burlingame, CA, USA). Complete protease inhibitor cocktail and BM Chemiluminescence Western Blotting Kit were obtained from Roche (Mannheim, Germany). Human gastric cancer MGC-803 cells and human gastric mucous epithelial GES-1 cells were available in the Cell Bank of Type Culture Collection of Chinese Academy of Sciences. Cell culture products and reagent were purchased from GIBCO. All the above chemicals were used without any further purification. Ultrapure water (Millipore Milli-Q grade) with a resistivity of 18.2 MΩ cm was used in all the preparations.

### Synthesis and characterization of Cdots and Cdots@PEI

The luminescent Cdots was synthesized by a one-step green route of microwave assisted pyrolysis method. Briefly, 2 g citrate and 0.01 g L-Trp was dissolved in 30 ml of ultrapure water, and stirring for 1 h to form a homogeneous solution in a 100 ml beaker. Then the beaker containing clear transparent solution was placed at the center of the rotation plate of a domestic microwave oven (700 W) and heated for 3 minutes. When cooled down to room temperature, the Cdots were isolated from the opaque suspension via centrifugation with 10,000 rpm which aims at removing carbon residual. Excessive citric acid and L-Trp were removed via repeated dialysis against deionized water using a low molecular weight cut-off membrane (1000 Da) for 2 days. Finally, different concentration can be got through rotary evaporation, which is used to remove water as it depends. The morphologies of the Cdots were detected using a transmission electron microscope (TEM, 2100F, JEOL, Japan), operating at an accelerated voltage of 200 kV. Photoluminescence (PL) spectra were measured on a Hitachi FL-4600 spectrofluorometer. UV–vis spectra were recorded with a Varian Cary 50 spectrophotometer (Varian Inc., Palo Alto, CA, USA). X-ray diffraction (XRD) measurement was performed with a D8 Advance (Bruker AXS Corporation, Germany). Fourier transform infrared (FTIR) spectra were conducted on a Nicolet 6700 spectrometer (Thermo Electron Corporation, Madison, WI) using KBr pellets. X-ray photoelectron spectrum (XPS) was acquired with a Kratos Axis Ultra^DLD^ spectrometer (AXIS Ultra, Kratos Analytical Ltd, Japan) using a monochromatic Al Kα source (1486.6 eV). Zeta potential was completed using a NICOMP 380 ZLS Zeta potential/Particle sizer (PSS Nicomp, Santa Barbara, CA, USA) equipped with a He-Ne laser (λ = 633 nm).

### Quantum yield measurement

The quantum yield of the Cdots was determined using quinine sulfate in 0.1 M H_2_SO_4_ (quantum yield: 54%) as the standard sample. According the emission peak area and absorbance of Trp@Cdots and quinine sulfate, the QY of the Trp@Cdots could be calculated from Equation 1 below:

$$ {}_{\varphi_{\mathrm{sample}} = \frac{\mathrm{A}{}_{\mathrm{std}}}{{\mathrm{A}}_{\mathrm{sample}}} \times \frac{{\mathrm{F}}_{\mathrm{sample}}}{{\mathrm{F}}_{\mathrm{std}}} \times \frac{{{\mathrm{n}}_{\mathrm{sample}}}^2}{{{\mathrm{n}}_{\mathrm{std}}}^2} \times {\upvarphi}_{\mathrm{std}}} $$

Where *Φ*_*std*_ is the known quantum yield of the standard compound, *F*_*sample*_ and *F*_*std*_ are the integrated areas of fluorescence of the sample and standard in the emission region at 350–600 nm. *A*_*std*_ and *A*_*sample*_ are the absorbance of the standard and sample at the excitation wavelength (360 nm); n is the refractive index of solvent, for water the refractive index is 1.33, 0.1 M H_2_SO_4_ is 1.33. All samples were diluted to ensure the optical densities less than 0.10 measured by Varian Cary 50 UV–vis spectrophotometer to minimize re-absorption effects.

### Preparation of siRNA-Cdots@PEI complexes and agarose gel electrophoresis

Three couples of siRNA oligonucleotides (noted as Surv-1, Surv-2 and Surv-3, respectively) and a couple of non-silencing-siRNA as negative control (NC) oligonucleotides were chemically synthesized by Shanghai Genechem Co. All siRNAs were annealed with complementary antisense strands with 3′-dTdT overhangs. The siRNA duplexes are as follows:

- Surv-1: sense, 5′-CACCGCAUCUCUACAUUCATT (dTdT)-3′; antisense, 5′-UGAAUGUAGAGAUGCGGUGTT (dTdT)-3′.
- Surv-2: sense, 5′-GAAGCAGUUUGAAGAAUUATT (dTdT)-3′; antisense, 5′-UAAUUCUUCAAACUGCUUCTT(dTdT)-3′.
- Surv-3: sense, 5′-GGUCCCUGGAUUUGCUAAUTT (dTdT)-3′; antisense, 5′-AUUAGCAAAUCCAGGGACCTT(dTdT)-3′.
- NC: sense, 5′-UUCUCCGAACGUGUCACGUTT(dTdT)-3′; antisense, 5′-ACGUGACACGUUCGGAGAATT(dTdT)-3′.

The Cdots-based complexes were formed by electrostatic interactions between positively charged PEI and the negatively charged Cdots, and then the negatively charged phosphate backbone of siRNA attaching to the Cdots@PEI complexes, resulting the siRNA loaded siRNA-Cdots@PEI complexes. The Cdots@PEI and siRNA-Cdots@PEI complexes were purified by gel filtration over a Sephadex G-50 column equilibrated with 10 mM NaCl to remove un-adsorbed PEI or siRNA. The electrophoretic mobility of the siRNA-Cdots@PEI complexes was determined by 1% agarose gel in 1× TBE buffer with a constant voltage of 120 V for 20 min. The siRNA loading amount was determined by measuring the absorption at 260 nm using the relation 1OD duplex = 3.0 nmols after subtracting the absorbance contributed by Cdots@PEI at the same wavelength. In aqueous solution of pH 7.4, the concentration of siRNA was determined to be about 15 nM when the concentration of Cdots@PEI was 100 μg/ml. All data when used for siRNA-Cdots@PEI complexes were expressed as 100 μg/ml Cdots@PEI with about 15 nM siRNA, unless otherwise specified.

### Cell culture and MTT assay

Human gastric cancer MGC-803 cells and human gastric epithelial GES-1 cells were available in the Cell Bank of Type Culture Collection of Chinese Academy of Sciences. All the cells were cultured in Dulbecco’s modified Eagle’s medium (DMEM) plus 10% (vol/vol) fetal bovine serum (Gibco) and penicillin-streptomycin (100 U/ml to 0.1 mg/ml) and incubated in a humidified incubator containing 5% CO_2_ at 37°C. MTT assay was carried out to investigate the cytotoxicity of Cdots and Cdots@PEI. MGC-803 and GES-1 cells were first seeded to 96-well plates at a seeding density of 5 × 10^3^ cells per well in 100 μl complete medium, which was incubated at 37°C for 24 h. Then the culture medium in each well was replaced by 100 μl fresh complete medium containing serial concentrations of Cdots and Cdots@PEI. After incubation for 24 h, the medium was replaced with 150 μL fresh medium containing 15 μl MTT (5 mg/ml in PBS) and incubated for another 4 h. Afterwards, the culture medium with MTT was removed and 150 μl/well of DMSO was added, followed by shaking for 10 min at room temperature. The absorbance of each well was measured at 490 nm using a standard micro plate reader (Scientific Multiskan MK3, thermo, USA). The cell viability was calculated according to the equation: cell viability = (OD_490 nm_ of the experimental group/OD_490 nm_ of the control group) × 100% and the cell viability of control group was denoted as 100%.

### *In vitro* siRNA transfection and cellular uptake of siRNA-Cdots@PEI complexes

Before transfection, the MGC-803 cells were seeded in 6-well plates and the appropriate transfected number of cells is based on the fact that confluent of cells achieve to 30% to 50% at the time of transfection. Each sample prepared siRNA oligo-Cdots@PEI complexes are as follows: A. siRNA oligo stock solution was diluted to 1 μM before transfection. Then 110 μl 1 μM of siRNA oligo was added to 200 μl serum-free DMEM and mix gently at room temperature for 5 min. B. After incubated for 5 min, 100 μl Cdots@PEI are taken into diluted siRNA oligo (mentioned in a.) with immediate shaking (using a scroll instrument or pipetting more than 10 s). After mild centrifugation, the solution needs to stand still at room temperature for 10 min, to allow the effective formation of siRNA oligo-Cdots@PEI complexes. C. While it is incubated, the medium in the cell culture plates need to be refreshed. Each well was added 1.8 ml of complete medium (containing 10% serum and antibiotics). The siRNA oligo-Cdots@PEI complexes were dropped into each well containing cells and the medium. Gently shake the culture plate after mixing. Complete medium can be changed after 4–6 h transfection. Scrambled siRNA with the transfection reagent of Cdots@PEI was used as the nontargeting control. After incubation with different transfection complexes for 48 h, approximately 2 × 10^5^ MGC-803 cells were collected from each sample and then subjected to quantitative reverse transcription-PCR (qRT-PCR) and Western blot analysis to determine the silencing efficiency against Survivin gene and Survivin protein expression. For evaluation of cellular uptake of siRNA-Cdots@PEI complexes, we tracked the cellular internalization of Cdots-PEI, Cy3-labelled siRNA, or Cy3-siRNA-Cdots@PEI in MGC-803 cells. The cells were plated on 14 mm glass coverslips and allowed to adhere for 24 h. After co-incubation with Cdots-PEI, Cy3-labelled siRNA, or Cy3-siRNA-Cdots@PEI for different times, the cells were washed twice with PBS sufficiently and fixed with 4% paraformaldehyde. Confocal fluorescence images were captured with a TCS SP5 confocal laser scanning microscopy (Leica Microsystems, Mannheim, Germany). Blue and red fluorescence images were acquired using DAPI-specific (excitation, 340–380 nm; emission, 450–490 nm) and Cy3-specific (excitation, 515–560 nm; emission, > 590 nm) sets of filters, respectively.

### Survivin expression assay by qRT-PCR analysis

In qRT-PCR experiment, the total RNA was extracted from transfected MGC-803 cells using the TRIzol reagent (Invitrogen, America) according to the manufacturer’s instructions. A total of 1 μg of RNA was transcribed into cDNA using random primers and M-MLV reverse transcriptase (Promega). The cDNA templates were stored at −20°C. The qRT-PCR was performed in a final volume of 25 μl containing 12.5 μl of Hieff™ qPCR SYBR® Green Master Mix (Yeasen, Shanghai), and 1 μl of each 10 μM primer, and 1 μl of 1:10-diluted cDNA products. The PCR amplification was carried out in a Bio-Rad iQ5 with one cycle at 95°C for 5 min, followed by 30 cycles at 95°C for 30 sec, at 67°C for 30 sec, and at 72°C for 1 min, and finally at 72°C for 5 min. GAPDH was chosen as the endogenous control in the assay. The following PCR primers were used: GAPDH primers, forward: 5′-CCACCCATGGCAAATTCCATGGCA-3′, reverse: 5′-TCTATCTAGACGGCAGGTCAGGTCCACC-3′; Survivin primers, forward: 5′-GTGAATTTTTGAAACTGGACAG-3′, reverse: 5′-CCTTTCCTAAGACATTGCTAA-3′.

### Western blot analysis

MGC-803 cells were lysed at 72 h after the transfection using RIPA lysis buffer (20 mM Tris, pH 7.5, 150 mM NaCl, 1% Triton X-100, 2.5 mM sodium pyrophosphate, 1 mM EDTA, 1% Na_3_VO_4_, 0.5 μg/ml leupeptin, and 1 mM phenylmethanesulfonyl fluoride) in the presence of complete protease inhibitor cocktail (Roche Diagnostics). The homogenate was then subjected to 10,000 rpm centrifugation for 10 min at 4°C. All the above procedures were performed in ice bath. The protein concentration was determined using BCA (bicinchoninic acid) protein assay kit (Beyotime Biotech, Jiangsu, China) and store at −20°C. The cell extracts (20 μg total proteins) were mixed with four times loading buffer (16% glycerol, 20% mercaptoethanol, and 2% SDS, and 0.05% bromophenol blue) (3:1, sample/loading buffer) and boiled for 5–7 min at 100°C. The samples were then subjected to 12% sodium dodecyl sulfate poly-acrylamide gel electrophoresis (SDS-PAGE) at 120 V for 1 h and then transferred onto 0.45 mm polyvinylidene difluoride membrane (PVDF, Immobilon-P 0.45 μm, Millipore, Billerica, MA), using a semi-dry system (Biocraft, Tokyo, Japan) at 300 mA for 150 min. Membranes were blocked with Tris-buffered saline containing 0.1% Tween 20 and 5% dry skim milk powder and then incubated with rabbit anti-human survivin antibody (1:1000, Epitomics) and rabbit anti-human β-actin antibody (1:2500, Epitomics) at 4°C overnight with a gentle shaking. The next day, after four 5 min washes with TBST buffer, the bolts were then incubated with horseradish peroxidase (HRP)-conjugated secondary antibody (goat anti-rabbit IgG, 1:2500, Epitomics) for 1 h at room temperature. Antibody binding was detected by enhanced chemiluminescence (BM Chemiluminescence Western Blotting kit, Roche) and autora-diography (Kodak X-OMAT; Kodak, Rochester, NY).

### Apoptosis assay by Annexin V-FITC and propidium iodide (PI) staining

The apoptotic and necrotic cells were analyzed by Annexin V/PI apoptosis detection Kit (Yeasen) according to the manufacturer’s protocol. In brief, MGC-803 cells were seed in 6-well plates at 5 × 10^4^ cells/well for 24 h before co-incubated with Surv-1-Cdots@PEI, Surv-2-Cdots@PEI, Surv-3-Cdots@PEI, NC-Cdots@PEI, and Cdots@PEI complexes, respectively. The cells incubated with complete medium only were set as blank control. After 48 h incubation, the cells were harvested, washed with PBS and re-suspended in 200 μL of binding buffer containing 5 μL Annexin V and 10 μL PI. After incubation in dark at room temperature for 15 min, 400 μL of binding buffer was added to each sample, and the cells were immediately analyzed by FACSCalibur (BD Biosciences, Mountain View, CA). The data analysis was performed with Flow Jo 7.6 software. Positioning of quadrants on Annexin-V/PI plots was performed to distinguish living cells (Annexin V^−^/PI^−^), early apoptotic cells (Annexin V^+^/PI^−^), late apoptotic/necrotic cells (Annexin V^+^/PI^+^).

### Cell cycle analysis

Cell cycle analysis was performed using Flow cytometry. After the incubation of MGC-803 cells with Surv-1-Cdots@PEI, Surv-2-Cdots@PEI, Surv-3-Cdots@PEI, NC-Cdots@PEI, and Cdots@PEI complexes for 48 h, respectively, cells were harvested, washed with PBS and fixed overnight in 70% ethanol at −20°C. Then the cells were washed with PBS and stained with 50 μg/ml PI and 100 μg/ml RNase A for 30 min in the dark at room temperature. The cell cycle phase distribution was acquired by FACSCalibur and G_1_, S, and G_2_/M populations were quantified using FlowJo 7.6 software.

## Result and discussion

### Synthesis and characterization of Cdots

**Figure 2:**
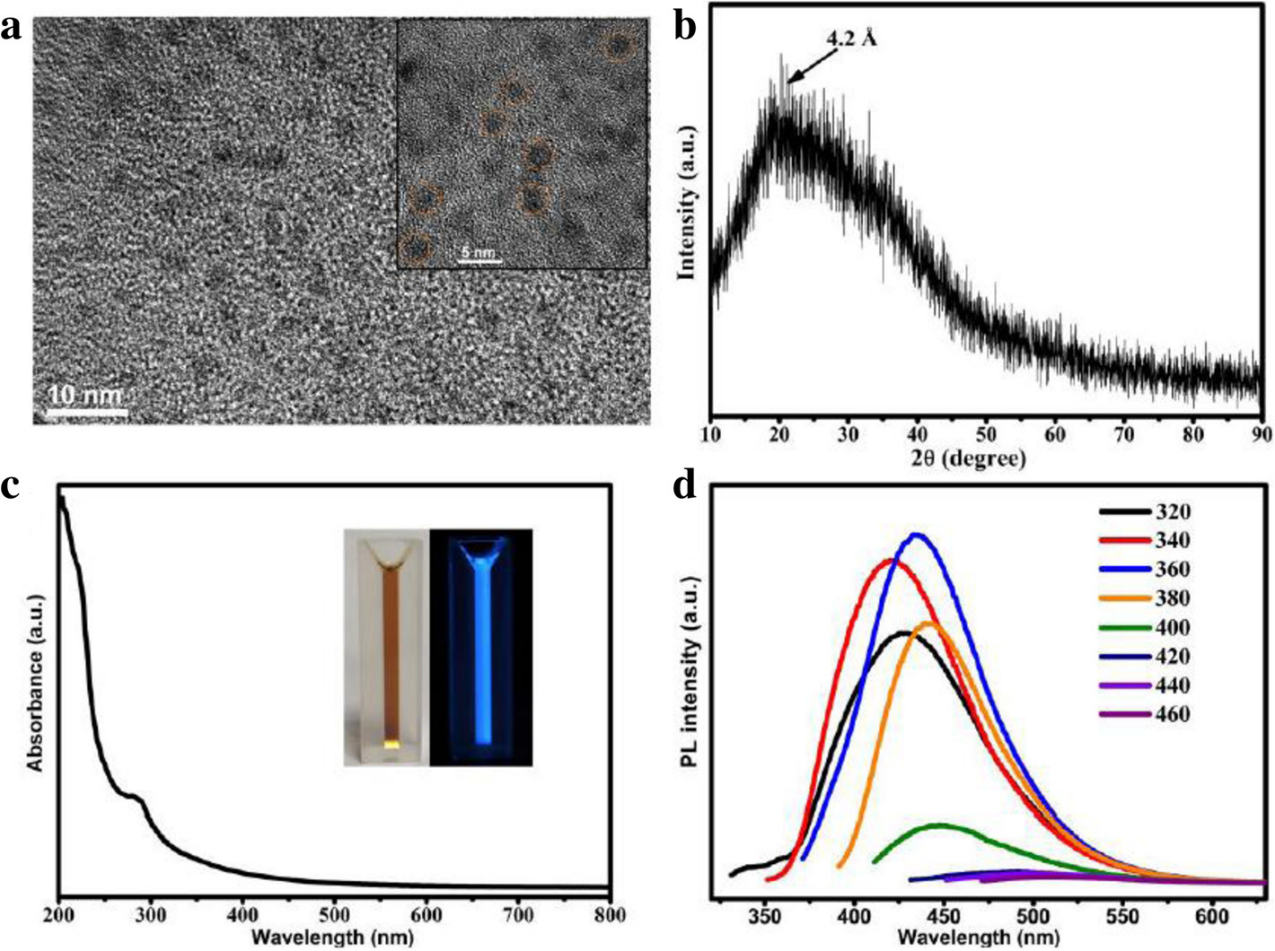
**Characterization of the as-synthesized Cdots (a) HRTEM images and inset is the zoom of particles. (b)** XRD pattern of the Cdots. The spherical Cdots are marked by circles. **(c)** UV–vis absorption spectrum of the Cdots, insets are digital photos of the Cdots dissolved in water under white-light (left) and UV (365 nm) excitation (right). **(d)** PL spectra of the Cdots when excited at different wavelengths from 320 to 460 nm in a 20 nm increment.

**Figure 3:**
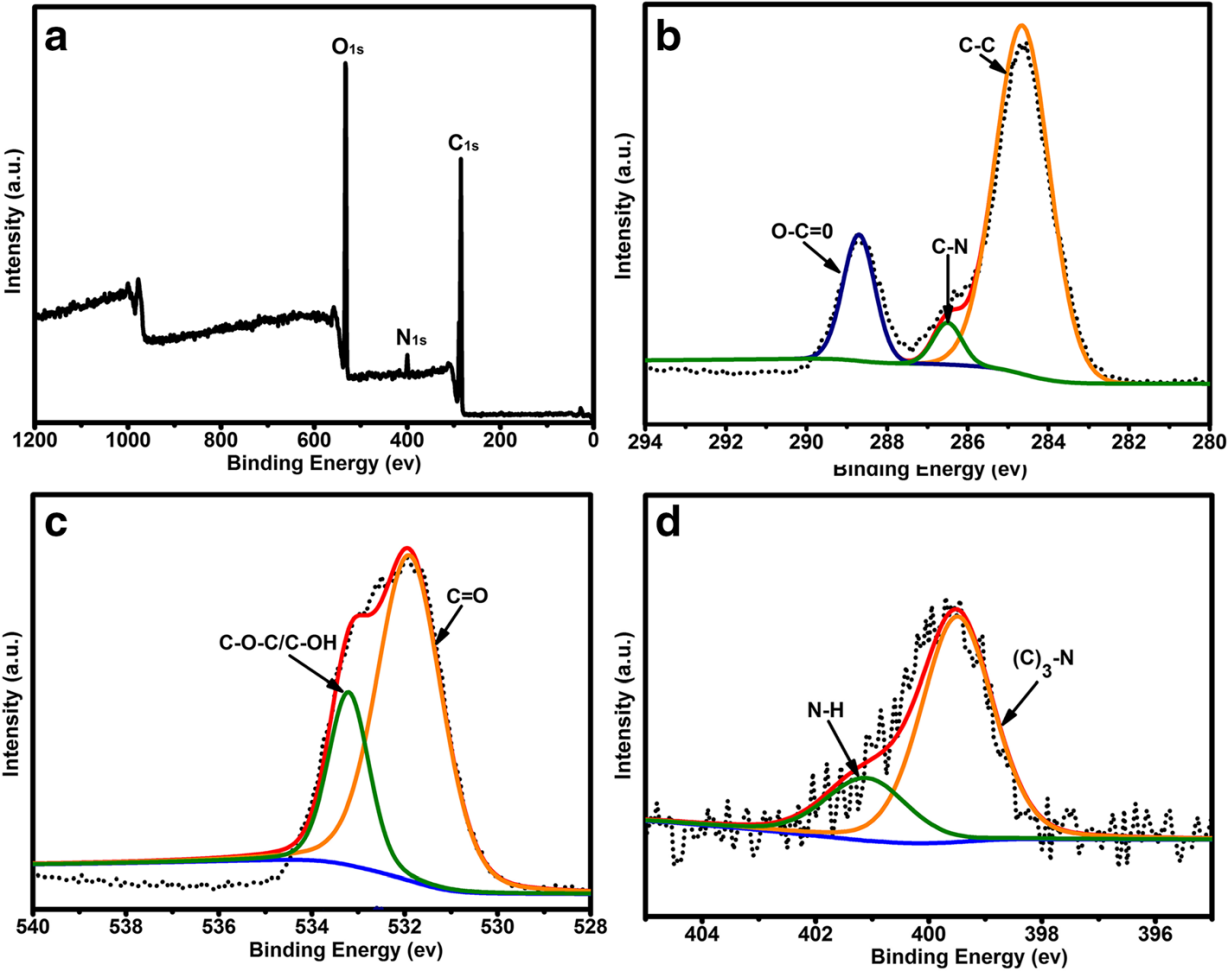
**XPS spectra of the Cdots. (a)** Survey spectrum of the Cdots with three major peaks of carbon, oxygen, and nitrogen. XPS high resolution survey spectra of **(b)** C1s, **(c)** O1s, and **(d)** N1s region of Cdots.

The morphology and structure of the Cdots were characterized by HRTEM and XRD. The HRTEM images indicate that the Cdots have outstanding uniform sizes and spherical shape, with an average diameter of about 2.6 nm (Figure 2a). The XRD pattern measured for Cdots shows a broad peak located at 2θ of around 20°(d = 4.2 Å), which is consistent with the (002) lattice spacing of Graphite, meanwhile, the larger interlayer spacing of 4.2 Å compared to that of bulk graphite which is about 0.33 nm might have resulted from the poor crystallization [7]. As shown in Figure 2c insets, the final product of Cdots is brownish in aqueous solution and emitted intensive blue luminescence under excitation of 365 nm UV light. The UV–vis absorption spectrum of the Cdots shows a clear an absorption feature at about 280 nm, which is ascribed to the function of aromatic rings of Tryptophan. The emission of the Cdots depends on the excitation wavelength. The Cdots show the strongest blue fluorescence under the excitation wavelength of 360 nm, with the highest quantum yield of 20.6%. XPS measurement was performed for the characterization of surface states (Figure 3). Three bands of the XPS survey spectrum at around 531.5, 400.0, and 284.5 eV represent O1s, N1s, and C1s, respectively, which indicating the atomic ratio of O/N/C is 54.6/3.6/41.8 as calculated from the survey spectrum [7,10]. The C1s core level spectrum can be deconvoluted into three contributions at 284.6, 285.2, and 289.0 eV, which are associated with carbon in the states of C-C, C-N, and O-C = O, respectively [7,9]. The O1s peaks at 532.0 and 533.2 eV can be assigned to oxygen in the form of C = O and C-O-C/C-OH, respectively [7,12]. The N1s peaks at 399.5 and 401.2 eV suggest that nitrogen exists mostly in the forms of (C)_3_-N and N-H, respectively [7,13]. Furthermore, Fourier transform infrared (FTIR) spectral measurement was conducted to determine the surface state of Cdots. As shown in Additional file 1: Figure S1, the as-synthesized Cdots show a main absorption band of O-H/N-H stretching vibration from 3630–2820 cm^−1^ and the existence of carbonyls (C = O) at 1717 cm^−1^. The bending vibration of C-O/C-N band and stretching peak of the C-O-C bond appear at 1400 cm^−1^ and 1194 cm^−1^, respectively [9,16,17]. In brief, the as-synthesized Cdots are rich in oxygen and possess mounts of hydroxyl/amine and carboxyl groups, which is useful for further modifications and biological applications.

### Formation and characterization of Cdots@PEI and siRNA-Cdots@PEI complexes

**Figure 4:**
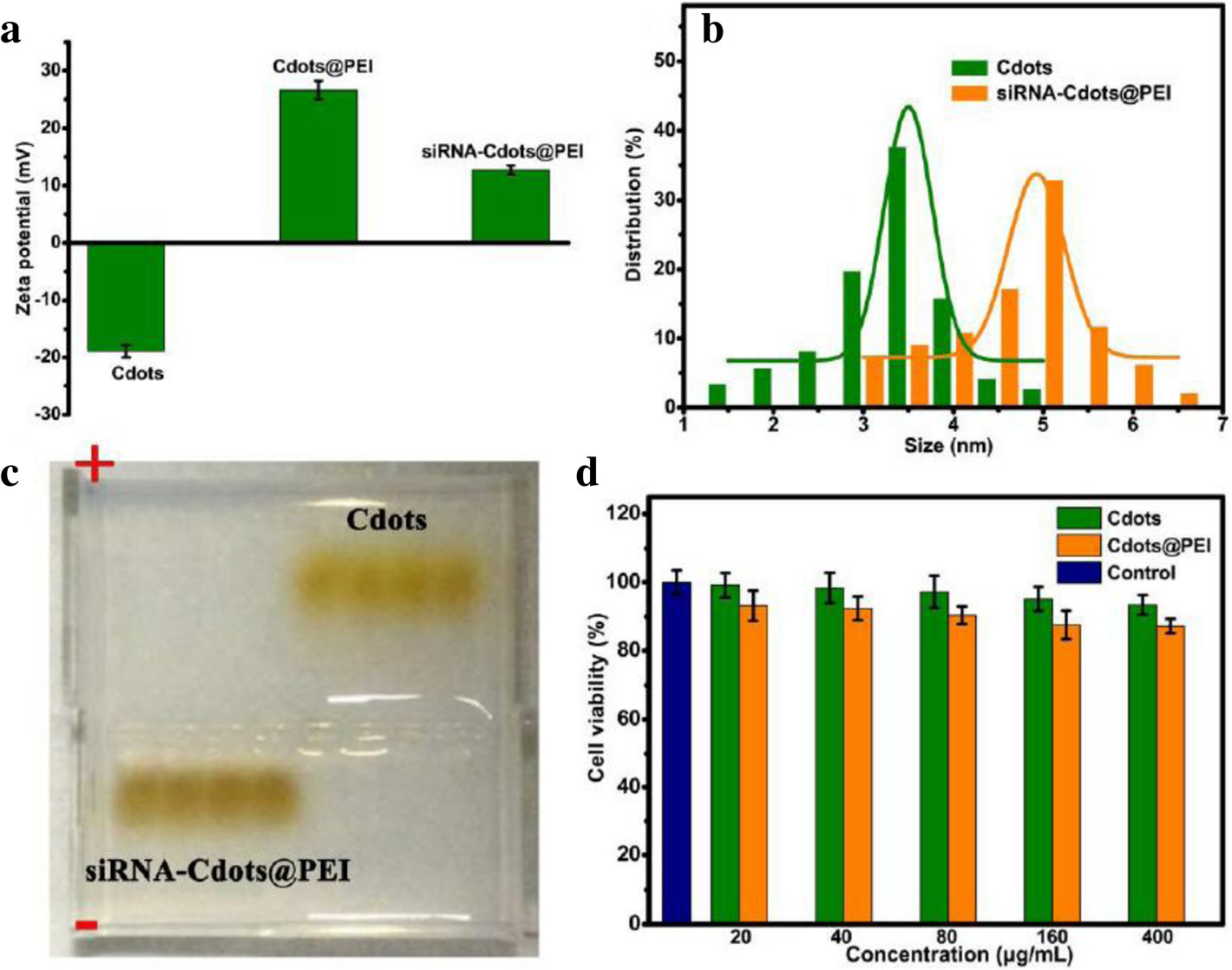
**Characterization of the Cdots@PEI and siRNA-Cdots@PEI complexes. (a)** Zeta potential of the Cdots, Cdots@PEI and siRNA-Cdots@PEI complexes. **(b)** Hydrodynamic diameters and **(c)** agarose gel electrophoresis analysis of the Cdots and siRNA-Cdots@PEI complexes. There were four paralleled loadings of each example at same time. **(d)** Cell viability of MGC-803 cells after treatment of different concentrations of Cdots and Cdots@PEI complexes.

The suspension of as-synthesized Cdots was highly stable in aqueous solution, with a zeta potential of −18.9 ± 1.2 mV at pH 7.4, imparting sufficient colloidal stability to the Cdots. Undoubtedly, it’s necessary to further exchange exterior charge of the Cdots for the siRNA delivery. So we introduce PEI, which have been reported to be individually considered as nonviral gene carriers with a capability of forming stable complexes by electrostatic interactions with nucleic acids. The zeta potentials of the as-prepared Cdots@PEI and siRNA-Cdots@PEI complexes were 26.6 ± 1.6 and 12.7 ± 0.8 mV, respectively, indicating the successful attachment of siRNA and PEI to the Cdots (Figure 4a). It is noteworthy that even after the load of siRNA; the positively charged siRNA-Cdots@PEI complexes could be conducive to intracellular delivery. The hydrodynamic particle size of siRNA-Cdots@PEI complexes was 4.7 ± 0.8 nm, which was a little larger than that of Cdots (3.9 ± 0.3 nm) (Figure 4b). The results of zeta potentials and hydrodynamic diameters indicated the favorable dispersibility of Cdots@PEI and siRNA-Cdots@PEI complexes in aqueous solution. Furthermore, agarose gel electrophoresis assay was taken to evaluation of capability of the Cdots@PEI complexes. With the attachment of negative charged siRNA, the surface positive charge of siRNA-Cdots@PEI complexes was neutralized and density of the complexes was raised, which caused the opposite direction of migration and the lower electrophoretic mobility when compared with Cdots (Figure 4c). Before the applications of the Cdots-based nanocarrier, the cell toxicity of Cdots and Cdots@PEI complexes was evaluated using MTT assays. Figure 4d showed the comparative viability of the cells incubated with Cdots and Cdots@PEI. It was found that Cdots induced no change in the cell viability when the dose was up to 400 μg/ml, suggesting their excellent biocompatibility. Previous reports have shown that the high content of cationic amine groups in branched polymer PEI mediated the substantial cytotoxicity [29]. The cell viability of MGC-803 cells was not seriously influenced by the addition of Cdots@PEI complexes, as it only slightly decreased to 87.2%. In this regard, the reduced cytotoxicity of Cdots@PEI can be explained by the reduced amounts of primary amine groups exposed at the surface of Cdots@PEI complexes.

### Intracellular delivery of siRNA by Cdots@PEI complexes

**Figure 5:**
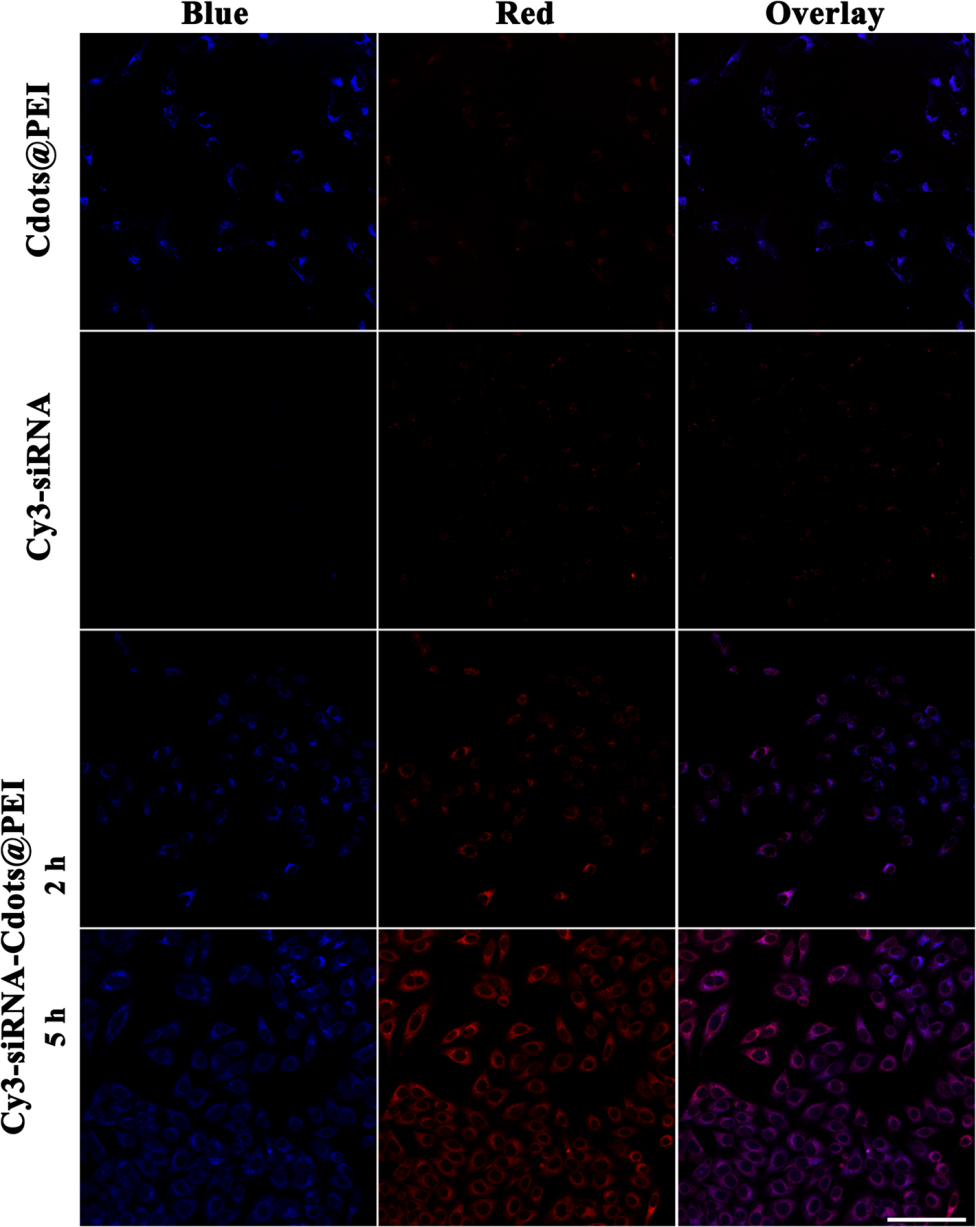
**Confocal laser scanning microscopic images of MGC-803 cells incubated with Cdots@PEI or Cy3-siRNA for 2 h, Cy3-siRNA-Cdots@PEI complexes for 2 and 5 h.**

As known to all, the negatively charged siRNA alone was inhibited from cellular uptake due to its inability to cross cellular membranes [24,25]. Herein, the positively charged siRNA-Cdots@PEI complex was proposed to enhance the cellular uptake and delivery of siRNA intracellularly. The resulting complex was used to transfect MGC-803 cells at pH 7.4 for 2 and 5 h, and the cells treated with Cdots or Cy3-labeled siRNA were set as controls. As a new fluorescent nanoprobe, the application of Cdots for bioimaging and biolabeling in cancer cells was evaluated. Fluorescence images were acquired using DAPI- and Cy3-specific sets of filters for the blue and red channels of fluorescence, respectively. As shown in Figure 5, after incubation with the Cdots@PEI complexes for 2 h, bright blue fluorescence was distributed in the cytoplasm while dim red fluorescence was observed. Consistent with the above excitation-dependent fluorescence, the intensity of red fluorescence excited from green light was much weaker than that of blue fluorescence excited from UV light. As anticipated, even after incubation with Cy3-siRNA for 5 h, only a marginal level of red fluorescence was detected within the cells, suggesting the low quantity of free siRNA that has entered the cells. In contrast, the siRNA-Cdots@PEI complex was rapidly accumulated into the MGC-803 cells within 2 h. With time growing, blue fluorescence from Cdots and red fluorescence from siRNA increase significantly at a time dependent manner. Hence, it is conceivable that the positively charged Cdots@PEI complexes play an important role in the great enhancement of siRNA internalization. The results indicated that the fluorescent Cdots-based siRNA delivery system not only can be efficient nanocarrier but also intracellular distribution reporter.

### Gene silencing efficiency of siRNA-Cdots@PEI complexes

**Figure 6:**
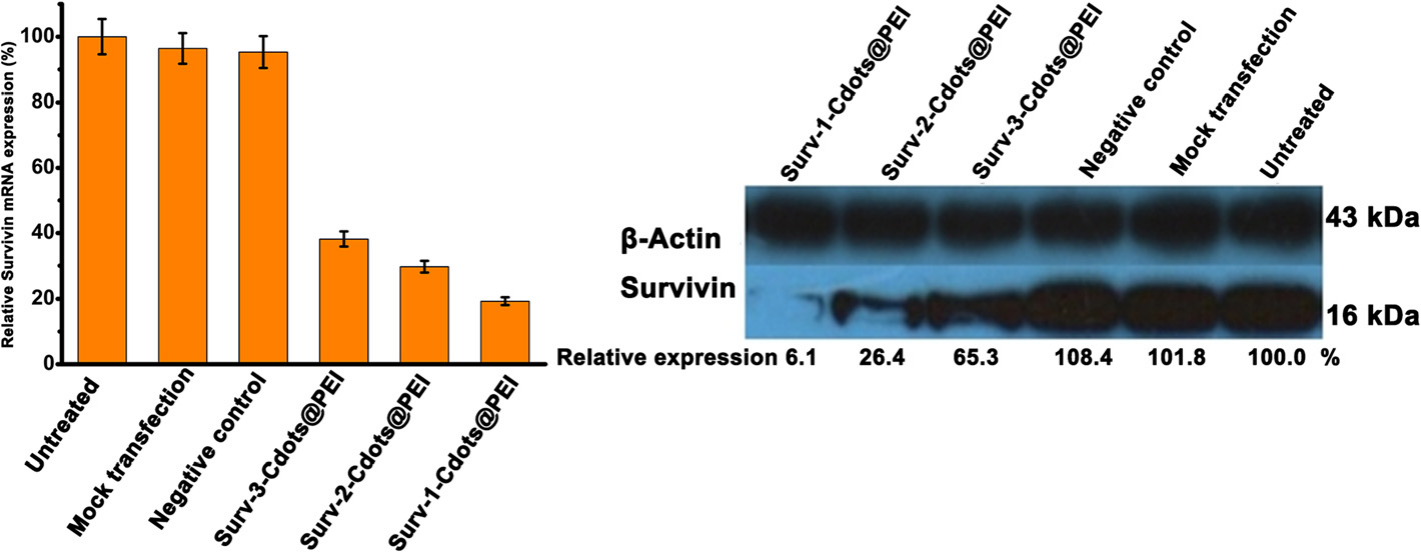
**Gene silencing efficiency of siRNA-Cdots@PEI complexes against Survivin at (a) mRNA and (b) protein expression level.**

Real-time PCR and Western blot assay was performed to evaluate the gene silencing efficiency of siRNA-Cdots@PEI complexes on the mRNA and protein level, respectively. The GAPDH was selected as a reference gene. The Cdots@PEI complexes functionalized with nontargeting scrambled siRNA served as a negative control. Compared with the blank control group (untreated), the expression level of Survivin mRNA was reduced to 19.3 ± 1.2%, 29.7 ± 1.8%, 38.2 ± 2.3%, 95.3 ± 6.9%, and 96.4 ± 8.7 when the cells were transfected with Surv-1-Cdots@PEI complexes, Surv-2-Cdots@PEI complexes, Surv-3-Cdots@PEI complexes, scrambled siRNA-Cdots@PEI complexes (negative control), and Cdots@PEI complexes (mock transfection), respectively (Figure 6a). The effect of Cdots@PEI complexes-based siRNA delivery on Survivin protein down-regulation was evaluated after 72 h of treatment. As shown in Figure 6b, the expression levels of protein of test group, Survivin in MGC-803 cells exhibited gradually down-regulation compared with the control groups, and the group of Surv-1-Cdots@PEI complexes exhibited the strongest inhibition effect on expression of Survivin protein, which corresponded well with the data given by qRT-PCR.

### Apoptosis assay and cell cycle analysis

**Figure 7:**
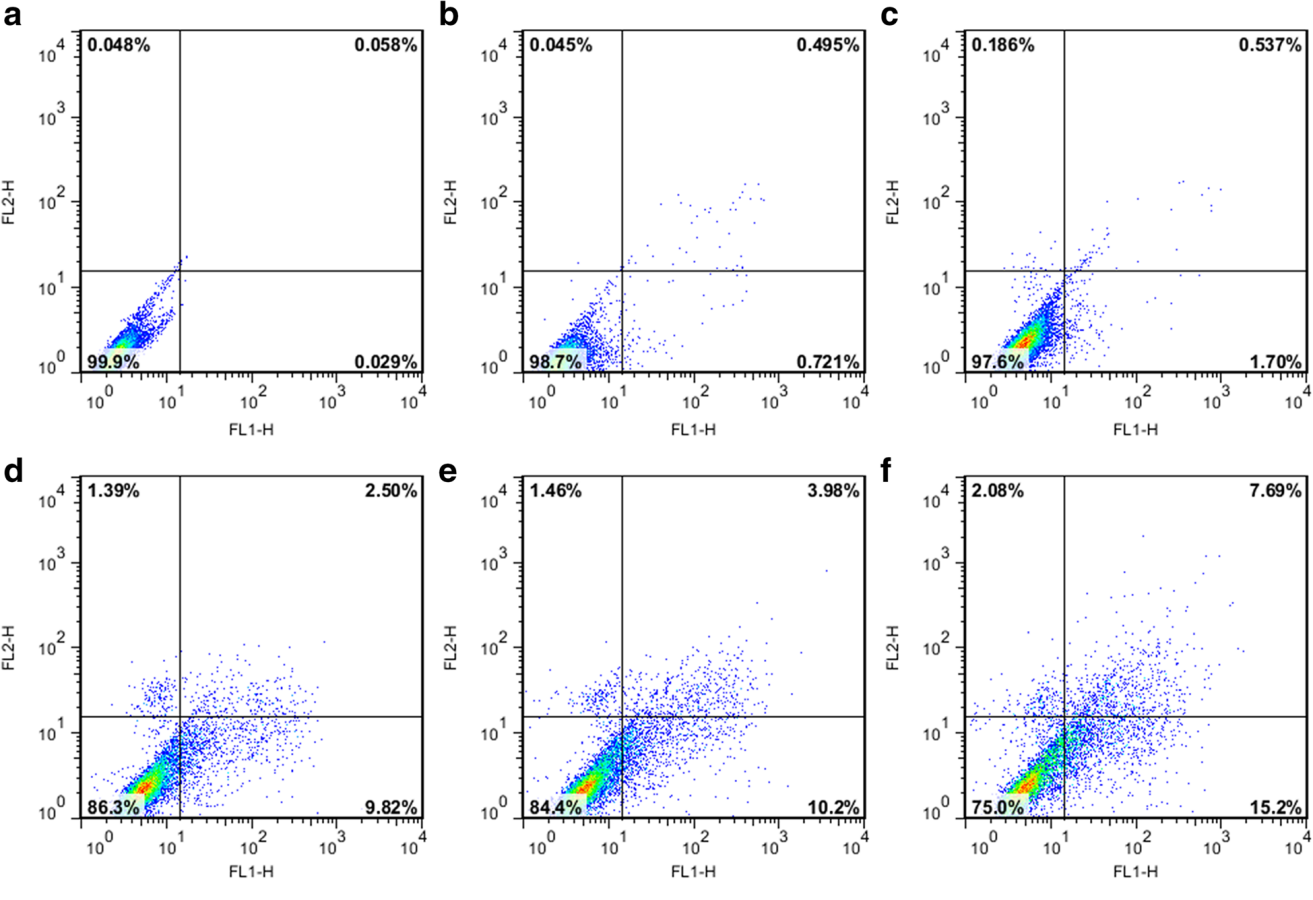
**Apoptosis induction in MGC-803 cells by siRNA-Cdots@PEI complexes mediated Survivin RNAi. (a-c)** Apoptosis analysis of untreated, mock transfection, and negative control groups, respectively. **(d-f)** Apoptosis analysis of MGC-803 cells treated with Cdots@PEI complexes-carried siRNA (Surv-3, Surv-2, and Surv-1) for 48 h, respectively.

**Figure 8:**
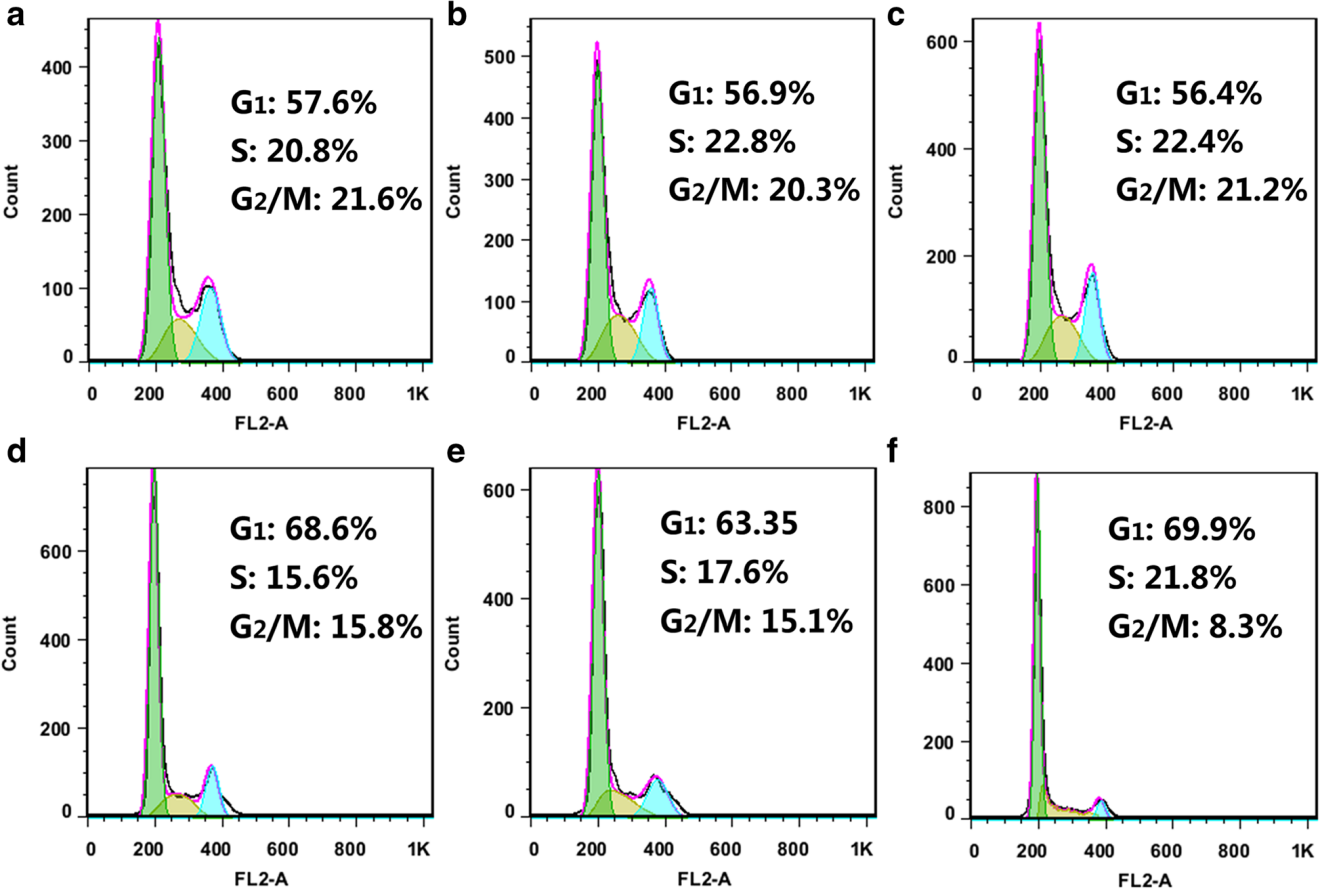
**Flow cytometry analysis of cell cycle arrest in MGC-803 cells induced by siRNA-Cdots@PEI complexes mediated Survivin downregulation. (a-c)** Cell cycle phase distribution of MGC-803 cells in untreated, mock transfection, and negative control groups, respectively. **(d-f)** Cell cycle phase distribution of MGC-803 cells treated with Cdots@PEI complexes-carried siRNA (Surv-3, Surv-2, and Surv-1) for 48 h, respectively.

In order to determine the secondary effects on the context of cell physiology by the downregulation of Survivin protein, the apoptosis levels and cell cycle distributions were evaluated based on flow cytometry analysis. Since Survivin inhibits apoptosis and improves the proliferation of cells, we hypothesized that the performed RNAi by siRNA-Cdots@PEI complexes in MGC-803 cells could induce apoptosis. Using Annexin V-FITC/PI double staining and flow cytometric analyses, rates of apoptosis were quantified as sums of early and late apoptotic cells. As shown in Figure 7a-c, the cells in blank (a), negative control (b), and mock (c) groups showed a large viable cell population with very fewer staining for early apoptotic, late apoptotic and necrosis cells, respectively. However, MGC-803 cells treated with Cdots@PEI complexes-carried siRNA (Surv-3, Surv-2, and Surv-1) for 48 h resulted in a shift from live cells to early apoptotic, late apoptotic, and dead cell populations (Figure 7d-f). Quantitative analysis of the data clearly demonstrated that Surv-3, Surv-2, Surv-1 traetment for 48 h resulted in 9.82%, 10.2%, and 15.2% early apoptotic cells; 2.50%, 3.98%, and 7.69% of late apoptotic cells, respectively. However, the dead cells also increased. It is widely accepted that cell cycle progression can be either negatively or positively regulated by a number of dynamically expressed genes. Previous studies have suggested that Survivin participates in multiple facets of cell division via controlling microtubule stability of the normal mitotic spindle [27,30]. Therefore, secondary effect was further extended to cell cycle analysis by propidium iodide (PI) staining-based flow cytometry cell cycle assay. As shown in Figure 8a-c, there was no significant change in cell cycle distribution in the cells treated with Cdots@PEI complexes (mock group) or scrambled siRNA-Cdots@PEI complexes (negative control group) when compared with the untreated cells (untreated group). However, the cells treated with Cdots@PEI complexes-carried siRNA (Surv-3, Surv-2, and Surv-1) demonstrated an increase in cell population in G_1_ phase, indicating that knockdown of Survivin could block the G_1_/S transition (Figure 8d-f). Therefore, we hypothesized that the critical role of Survivin in early cell cycle entry would explain cell cycle arrest MGC-803 cells when Survivin was down-regulated.

## Conclusion

In summary, the Cdots-based and PEI-adsorbed complexes both as imaging agent and siRNA nanocarrier have been developed for Survivin siRNA delivery. The Cdots were prepared *via* one-step microwave assisted approach with citric acid as carbon source and tryptophan as passivation agent and nitrogen source. The as-synthesized Cdots exhibited excellent water dispersibility, biocompatibility, and high quantum yield. In the elaborately fabricated complexes of siRNA-Cdots@PEI, Cdots acted as a nanocarrier and a fluorescent indicator, while the positively charged PEI acted as the ties attaching negative charged siRNA to Cdots. Furthermore, the confocal fluorescence images indicted the cellular uptake of siRNA-Cdots@PEI complexes, and subsequently qRT-PCR and Western blot analysis confirmed the successfully entrance of siRNA into MGC-803 cells and superior gene silencing efficiency. Importantly, the siRNA-Cdots@PEI complexes, which target Survivin gene, can induce apoptosis and cell cycle arrest in G_1_ phase inhuman gastric cancer cells MGC-803. The resulting Cdots-based delivery system may be used to advance the field of siRNA therapeutics.

## Supporting information

Supporting information is available from the XX Online Library or from the author.

## Additional file

Additional file 1: Figure S1. FTIR spectra of tryptophan, citric acid, and the Cdots.
