# Supplementary material for: Fluorescent carbon dots as an efficient siRNA nanocarrier for its interference therapy in gastric cancer cells
Source: J Nanobiotechnology. 2014 Dec 30;12:58. doi: 10.1186/s12951-014-0058-0 (PMC4304159; doi:10.1186/s12951-014-0058-0)
Supplement: Additional file 1: Figure S1. — FTIR spectra of tryptophan, citric acid, and the Cdots. [file 12951_2014_58_MOESM1_ESM.docx]

**Fluorescent carbon dots as an efficient siRNA nanocarrier for its interference therapy in gastric cancer cells**

Qing Wang^1,2†^ ,Chunlei Zhang^2^† Guangxia Shen^2*^, Huiyang Liu^2^, Hualin Fu^2^, and Daxiang Cui^1,2*^

*^1^School of Life Sciences and Biotechnology, Shanghai Jiao Tong University, Shanghai 200240, China.*

*^2^Institute of Nano Biomedicine and Engineering, Key Laboratory for Thin Film and Microfabrication Technology of the Ministry of Education, Department of Instrument Science & Engineering, School of Electronic Information and Electrical Engineering, Shanghai Jiao Tong University, 800 Dongchuan RD, Shanghai 200240, China.*

*E-mail:* [*sxshen@sjtu.edu.cn*](mailto:sxshen@sjtu.edu.cn) *and* [*dxcui@sjtu.edu.cn*](mailto:dxcui@sjtu.edu.cn)

† These authors contributed equally to this work.


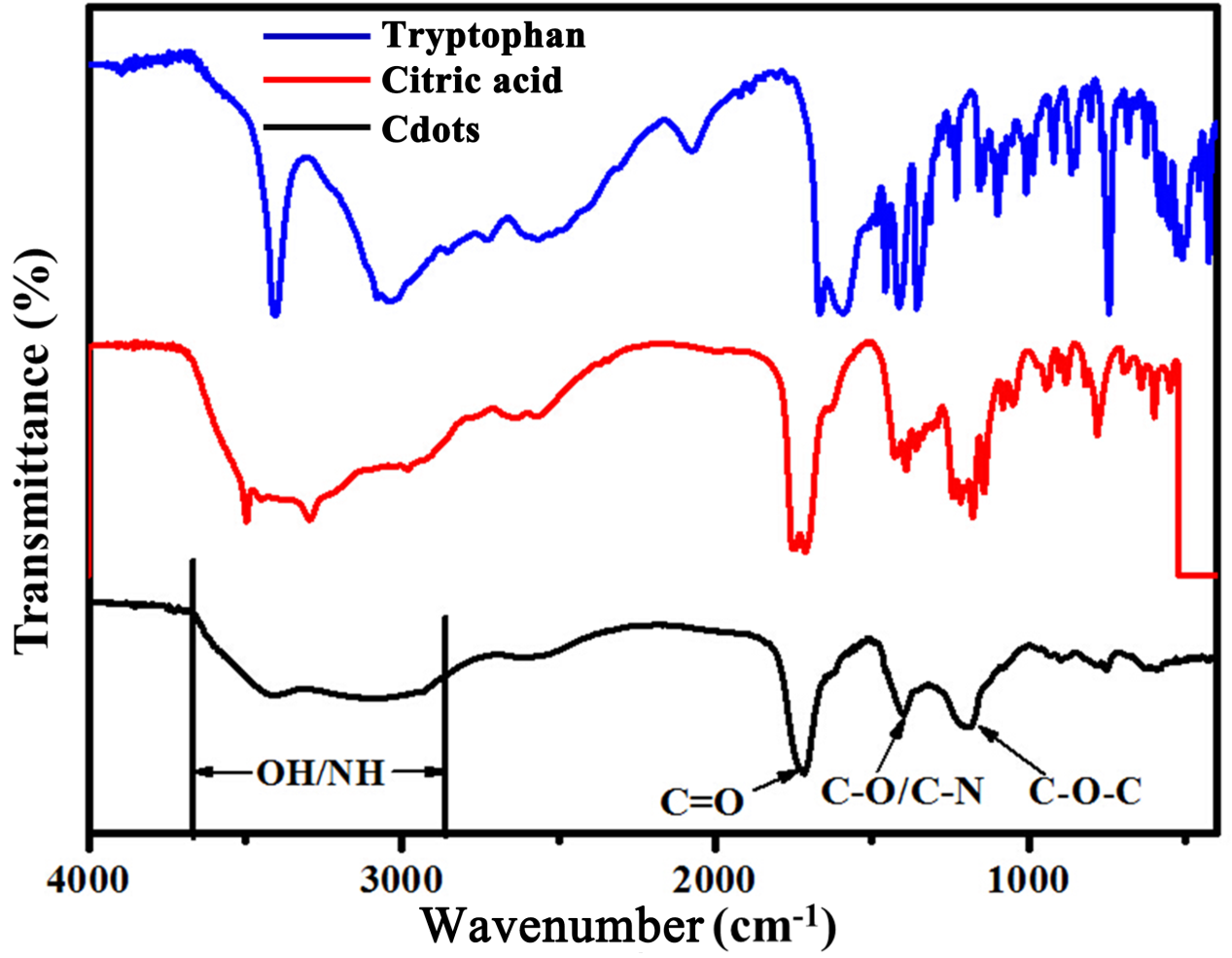


**Figure S1** FTIR spectra of tryptophan, citric acid, and the Cdots.
